# Population measurement for health systems

**DOI:** 10.1038/s41746-017-0004-2

**Published:** 2018-01-15

**Authors:** Bruce R. Schatz

**Affiliations:** Department of Medical Information Science, Carl R. Woese Institute for Genomic Biology, University of Illinois, Urbana, IL 61801 USA

**Keywords:** Risk factors, Health services, Signs and symptoms, Diagnosis, Respiratory tract diseases

## Abstract

How can health systems make good use of digital medicine? For healthcare infrastructure, the answer is population measurement, monitoring people to compute status for clustering cohorts. In chronic care, most effective is measuring all the time, to track health status as it gradually changes. Passive monitors run in the background, without additional tasks to activate monitors, especially on mobile phones. At its core, a health system is a “sorting problem”. Each patient entering the system must be effectively sorted into treatment cohorts. Health systems have three primary problems: Case Finding (which persons have which diagnoses), Risk Stratification (which persons are which status), and Care Routing (which persons need which treatments). The issue is then which measures can be continuously monitored at appropriate periodicity. The solutions of population measurement measure vital signs with passive monitors. These are input to predictive analytics to detect clinical values for providing care within health systems. For chronic care, complex vitals must be measured for overall status, such as oxygen saturation or gait speed. This enables healthcare infrastructure to support stratification, with persons placed into current levels of health status. Practical considerations for health systems influence implementation of new infrastructure. Case finding is more likely to be useful in urban settings, with barriers to entry based upon lower incomes. Care routing is more likely to be useful in rural settings, with barriers to entry based upon isolated geographies. Viable healthcare at acceptable quality and affordable cost is now possible for the range of geographies and incomes.

## Introduction

How can health systems make good use of digital medicine, especially of mobile monitors? The answer for healthcare infrastructure lies in population measurement, monitoring all of the people all of the time and computing their status to cluster into cohorts. There has been significant progress with data analysis of specific procedures^1^ for efficiency and efficacy, but little focus upon general problems of health systems.^2^ This is particularly true of management of major chronic conditions, especially cardiopulmonary conditions of older adults such as CHF (congestive heart failure) and COPD (chronic obstructive pulmonary disease), which are major costs for Medicare.

Population measurement improves when more persons are monitored with more features. In acute care, monitors are utilized during intense but sporadic episodes. In chronic care, it would be best to measure all the time, to track the health status as it gradually changes over time. Active monitors require patients do some task, such as take their temperature or use a home blood pressure monitor. Passive monitors run all the time in the background, without additional tasks to activate the monitors. Wearable devices fall into this category, for the ones that can be continuously worn, as do carried devices such as mobile phones.

Medical devices that are wearable typically require correct positioning, to achieve clinical accuracy. Such issues with compliance have prevented widespread usage by many patients, even with the best devices.^3^ Fitness devices that are wearable typically require compact formfactors, to achieve usage convenience. Limited battery size forces less frequent sampling, which limits measuring cardiopulmonary function. Capturing walking motion, for example, requires 20 samples per second, reflecting human biomechanics of gait cycles.^4^ Activity monitors used in clinical experiments achieve such rates,^5^ but fitness devices currently support far less. So wristbands cannot measure functional status, only steps for healthy individuals, with stepcounts not accurate for pulmonary patients.^6^

Population measurement for health systems can be implemented now with mobile phones. Even the cheapest smartphones, currently costing only $30, can easily measure walking motions with clinical accuracy.^6^ Ordinary smartphones simply carried during daily activities have sufficient sensors to measure function. For example, characteristic motions from smartphone sensors are sufficient to predict functional status of pulmonary disease.^7^ Mobile phones are nearly ubiquitous,^8^ even among older adults, and are also communications devices that can transmit sensor data to remote servers for deeper analytics. Future devices with wireless transmission may eventually supplant smartphones for population measurement, such as standalone smartwatches with multiple sensors or washable smartclothes with sensor grids.

## The problems of health systems

At its core, a health system is what computer scientists call a “sorting problem”. That is, each patient entering the system must be effectively sorted into treatment cohorts. A typical health system has hundreds of treatment guidelines, so each patient must be sorted into one or more categories, with the optimal combination of quality (efficacy) and cost (efficiency). The difficulty, of course, is maintaining high value with high volume. Accordingly, health systems have three primary problems with respect to population measurement: Case Finding (which persons have which diagnoses), Risk Stratification (which persons are which status), and Care Routing (which persons need which treatments).

*Case Finding* involves distinguishing at-risk patients from disease incidences, to enable early detection at population scale. That is, automatically determine whether a patient has a disease compared to a patient with the same demographics who does not have it. Within healthcare infrastructure, case finding provides possible candidates for early detection of chronic disease, which can be confirmed by physicians. Typically this requires distinguishing between mild cases and severe cases, thus identifying cohorts who would benefit from medical treatments. For example, with COPD, case finding involves distinguishing no COPD or mild COPD versus moderate or severe levels. Developing recognizers for finding cases requires testing patients who have been recently diagnosed, to support training for onset detection.

Such capacity is essential for cardiopulmonary diseases. For example, the US CDC estimates 16 M diagnosed cases of COPD, but that only half of actual cases are diagnosed. Despite this, the USPSTF (United States Preventative Services Task Force) recommends that general screen screening of asymptomatic adults is not effective.^9^ Current methods for case finding use quality-of-life questionnaires for screening purposes followed by limited spirometry,^10^ which place significant burdens on primary care while failing to scale to large populations. Passive monitors with ordinary smartphones could automatically perform case finding for entire populations, while routing potential cases into primary care for confirmatory diagnosis and prevention.

*Risk Stratification* involves determining the current health status, as clinical indication of disease severity to guide treatments. To be effective for population measurement, the chronic disease status must have a standardized delineation into discrete categories. For example, with pulmonary function, those patients without COPD can be treated differently than those with COPD, and those with status level 1 (mild) treated differently than those with status level 2 (moderate). Within healthcare infrastructure, risk stratification provides determination of health status with clinical accuracy corresponding to established methods, which can be physiologic or symptomatic. Developing recognizers for stratifying risk requires testing patients across the range of status levels, while adjusting for demographics variation.

With pulmonary disease, for example, the GOLD levels for COPD can be predictively modeled. The GOLD status levels are measured by medical devices called spirometers into which patients breathe for one second, recording into electronic medical records what amount of air is expired. These then measure via threshold ranges as mild to moderate, severe to very severe COPD,^11^ corresponding to GOLD levels 1/2/3/4. Patients at each level also have a characteristic motion,^12^ as they pause to catch their breath during walking, which can be detected by motion sensors in mobile phones.^7^ Status levels for CHF function are also standardized, but not as consistently,^13^ since based upon symptoms rather than physiology.

*Care Routing* involves treating the patient regarding their diagnosis and status, at an appropriate facility. A simple time-critical process is nurse triage, who consults with patients by telephone, quickly routing care to hospital, clinic, or home. Triage heuristic software enables nurses to ask questions then route care, where the digital medicine equivalent is phone software that can directly query patients before providing care advice. Complex versions utilize particular diagnoses for particular patients, with situational awareness provided by medical records from health systems and risk stratification from population measurement. This requires establishing standard levels of health status, which can be correlated to treatment guidelines. Such guidelines for health systems will typically differ based on both the text of diagnosis and the context of risk level. Corresponding treatments will vary from lifestyle at mild levels to medical at severe levels.

Complementing the treatment guidelines for overall care routing, health systems often support action plans for common conditions. These describe effective actions at progressive stages of chronic diseases. Thus they focus upon the symptoms that are actionable. Typically, early stages focus upon lifestyle changes in diet and exercise to slow progression of disease, while later stages focus upon medical interventions of drugs and surgery to maintain viable levels of functional status. For example, with the common condition of COPD, an action plan could recommend rehabilitation at early stages and intervention at later stages.^14^ The rehabilitation enables patients to pace themselves to maintain adequate lung capacity while walking, while the intervention enables patients to increase lung capacity with supplementary oxygen plus corticosteroids.

## The solutions of population measurement

A typical health system has 400 K patients, and each patient is typically in multiple treatment categories, say 2 or 3. This is dependent upon demographics, especially age and sex. So with 1000 typical treatments, each category will on average have 1000 patients. This problem definition reduces the system solution to a standard situation within statistical clustering, namely sorting 1 M objects into 1 K categories, with similar objects within each category. Accomplishing this effectively will require feature vectors with appropriate discriminations. Population measurement can be deployed effectively when sufficient features can be continuously monitored. Assessment periodicity depends on contextual factors beyond disease levels, such as personal symptoms and social stressors, in addition to medical history.

The problems of health systems can be reduced to what measures can be continuously monitored, for sufficient input to predictive analytics to compute clinical values relevant to providing care. The traditional method with acute care is to measure vital signs, which are indeed of clinical utility. It has long been established which values can be easily measured yet must be quickly remedied. Each of the four primary vital signs must exist within a narrow range of clinical values, for the person to be viable. This is true of body temperature and blood pressure, heart rate and respiratory rate. If any of these drops too low, or rises too high, the person is at severe risk in the short term.

Measuring vitals is a primary reason that acute care is so effective, both in diagnosis and especially in treatment. But little is known about typical values during daily living, so they are too simple to be effective for chronic care. There is also significant range of normal values in vital signs.^15^ Despite intriguing anecdotes, the only consistent measurements have been those where simple patterns predict clinical events. For example, there are wearable devices to detect atrial fibrillation,^3^ but heart monitors can predict heart attacks within a few minutes, not within a few months as needed for chronic management.

Thus, simple vitals are effective for acute care. But complex vitals are necessary for chronic care. Since “chronic” means “time”, a simple threshold is not sufficient to detect a particular status that puts a person at risk. What is needed is more complex, in two senses. First, the measurement itself must be composite, reflecting the overall status of the body, not just the status of an individual organ or system. Thus a composite measure such as oxygen saturation reflects there is a clinical problem, more often than pulse and respiratory rates. Oxygen saturation, the proportion of oxygen in the blood, combines the effects of the circulatory system and the respiratory system. It is a standard composite status measure in clinical practice,^16^ the complex vitals analogue of simple blood pressure.

Second, the pattern of the measure must be correlated to status. Oxygen saturation is most commonly used during a procedure to assess clinical risk. If the value measured by a pulse oximeter drops more than a specified amount, then the person is at risk and the procedure must be stopped. Availability of methods for continuously measuring oxygen saturation leads to the possibility of detecting complex patterns correlated with health status for chronic disease. For example, motion sensors in mobile phones can detect patterns of oxygen saturation for transition categories, based on standard deviation during walking tests for chronic patients.^17^ A transition category is intermediate between classes of cardiopulmonary function, e.g., between GOLD1 and GOLD2. Monitor alerting to care providers can focus on patients in transition, since changes in status correlate with the patients most likely to require changes in care. Eventually, future changes may be predictable, a month or a year in advance.

The complement to internal measures of oxygen saturation, are the external measures of walking patterns. If oxygen saturation can be considered the fifth vital sign,^18^ then gait speed could be considered the sixth vital sign,^19^ since other contenders such as pain cannot be easily classified. External walking reveals many aspects of internal function, as a standard textbook in internal medicine states:^20^ “Watching a patient walk is the most important part of the neurological examination. Normal gait requires that many systems, including strength, sensation, and coordination, do function in an integrated fashion.” The 6 min walk test is widely used to evaluate cardiopulmonary status.^21^ This involves walking back and forth in a corridor, as in hospital or clinic, continuing past when typical cardiopulmonary patients slow down due to lack of blood and breath.

The purpose of population measurement is *stratification*. The status categories are used to place each person into current classes of health levels. Each person exists within multiple classes, corresponding to multiple dimensions of health status. The goal of population management is to move patients from “less healthy” to “more healthy” classes, via treatments and lifestyles. Since chronic diseases change over time by definition, patients will move from class to class. But better management moves more persons to healthier categories. Note the classes are treatment centric, since their purpose is assigning “optimal” categories for healthcare transformation. Thus persons requiring similar treatments may not necessarily have similar diagnoses, as the classification scheme is based upon health status, not only disease identification.

Supporting such rational basis for healthcare infrastructure implies there can be systematic basis for treatment guidelines.^2^ This is analogous to existing health systems which implement action plans for disease classes. These action plans today are primarily based upon disease diagnoses, for diseases with systematic levels. For example, a lung disease, such as COPD, specifies actions for each GOLD level, while a heart disease, such as CHF, would have different action plans to correspond with different status levels. Having widespread population measurement, such as possible with passive monitors, enables establishment of a wide range of action plans. These would support population management across all major conditions, which can be clinically assigned with consistent status.

## Conclusions

Once passive monitors are deployed throughout the population for healthcare measurement, it becomes possible to establish healthcare infrastructure for chronic diseases. As noted, motion sensors in personal smartphones are the major catalyst for measuring health status. This is true for major cardiopulmonary conditions, such as CHF^22^ and COPD,^23^ where walk tests can measure health status for clinical decisions. Gait is also predictive of status for rheumatoid arthritis^24^ and common chronic conditions.^25^ Leveraging normal usage of carried phones can provide significant monitoring for entire populations, with sensor recording on phone clients and predictive analytics on remote servers.

For health systems to utilize new technologies for healthcare infrastructure, these must be seamlessly integrated into standard processes for primary care. Monitor status for routine care must become the new standard for population health.^26^ The recording software can be loaded onto personal smartphones during office visits, or offered via phone-based patient portals to electronic medical record systems. Both strategies leverage the trust in health systems, not generally true of healthcare apps where patient privacy often conflicts with commercial profitability. Point of contact diagnostics can be broadly supported when significant deviation from current status is detected. This point also provides teachable moments for clinical advice to patients, with targeted messages at relevant times. Additional information can then be solicited from the patient, for deeper context in providing treatments guided by situational analysis.

Practical considerations for health systems influence implementation of new infrastructure. Case finding is more likely to be useful in urban settings, where the barrier to entry is based upon lower incomes. Care routing is more likely to be useful in rural settings, where the barrier to entry is based upon isolated geographies. The State of Illinois has significant examples, for both types of barriers, as a large populated region including urban cities with lower incomes and rural towns with isolated geographies as exemplified by Chicago and by Peoria respectively. What plays in Peoria does not play in Chicago, and vice versa. But mobile phones have nearly complete penetration into both healthcare markets.

Passive monitors have great benefit for urban systems providing the safety net for lower income patients. The patients cannot afford much technology, but nearly all have mobile phones. Even with the safety net, they often cannot afford healthcare interactions, so passive monitors within healthcare infrastructure have great potential. The barrier to entry is affordability, rather than availability. Since interactions are infrequent, the focus is on case finding. Discovering that a patient has a condition, with early detection pre-diagnosis, can significantly improve care. Cases can be discovered early, and backed off from severe status. The clinics throughout the city region can target their limited care to those with positive identification of chronic cases. Respiratory diseases are endemic in these environments due to substandard housing, so COPD and asthma are prime candidates. The capability to automatically monitor large populations of lower income patients will have revolutionary implications for those most likely to be excluded from the health system. Even just continuous touching of each patient at low cost may be inclusive and therapeutic.

Passive monitors have great benefit for rural systems supporting small towns, isolated geographically although often middle class. It is no longer economic to support hospitals in small towns of tens of thousands of persons. The barrier to entry is availability, rather than affordability. The patients could afford hospital visits, but the systems cannot afford to maintain isolated facilities for this scale. Since mobile phones are ubiquitous in these populations, there is great potential for passive monitors within healthcare infrastructure. Since the interactions often require transportation, the focus is on care routing. Here health status could be determined on a daily basis, for each patient for each condition, then utilized to treat patients at appropriate levels of the provider pyramid. Beyond heuristic triage, each patient can be routed to home care or local emergency, to handle the vast majority of care interactions. For less common situations, the patient can be routed to regional clinics or central hospitals, but only when this care is necessary. The remote analytics would be performed at the central city site, while the local monitoring would be performed in the small towns. The continuing future of rural healthcare relies on successful implementation of such command and control systems.

New infrastructures represent the vanguard of viable healthcare. These could be funded via unique combinations of public-private funds, with federal and state governments for Medicare and Medicaid, supplemented by health systems and community donors. There is a path towards universal healthcare at acceptable quality and affordable cost for the range of geographies and incomes.
